# Supplementary material for: Broadly Effective ACE2 Decoy Proteins Protect Mice from Lethal SARS-CoV-2 Infection
Source: Microbiol Spectr. 2023 Jul 3;11(4):e01100-23. doi: 10.1128/spectrum.01100-23 (PMC10434153; doi:10.1128/spectrum.01100-23)
Supplement: Supplemental file 1 — Fig S1-S8. Download spectrum.01100-23-s0001.pdf, PDF file, 2.1 MB [file spectrum.01100-23-s0001.pdf]

Supplementary Materials for

Broadly effective ACE2 decoy proteins protect mice from lethal SARS-CoV-2 infection

Mengjia Lu[a,b], Weitong Yao[d], Yujun Li[e]*, Danting Ma[f], Zhaoyong Zhang[c], Haimin Wang[b,h], Xiaojuan Tang[a,b], Yanqun Wang[c], Chao Li[b], Dechun Cheng[b], Hua Lin[g], Yandong Yin[a,b]*, Jincun Zhao[c]*, Guocai Zhong[a,b,h,i,j]*

[a] State Key Laboratory of Chemical Oncogenomics, Guangdong Provincial Key Laboratory of Chemical Genomics, Peking University Shenzhen Graduate School, Shenzhen, Guangdong 518055, China.
[b] Shenzhen Bay Laboratory, Shenzhen, Guangdong 518132, China.
[c] State Key Laboratory of Respiratory Disease, Guangzhou Institute of Respiratory Health, First Affiliated Hospital of Guangzhou Medical University, Guangzhou, Guangdong 510182, China.
[d] Hubei JiangXia Laboratory, Wuhan, Hubei 430200, China
[e] Shenzhen University School of Medicine, Shenzhen, Guangdong 518000, China.
[f] Tianjin Medical University Chu Hsien-I Memorial Hospital, Tianjin 300134, China.
[g] Biomedical Research Center of South China, Fujian Normal University, Fuzhou, Fujian 350117, China.
[h] Current Affiliation: Horae Gene Therapy Center, University of Massachusetts Chan Medical School, Worcester, MA 01605, USA.
[i] Current Affiliation: RNA Therapeutics Institute, University of Massachusetts Chan Medical School, Worcester, MA 01605, USA.
[j] Current Affiliation: Department of Biochemistry and Molecular Biotechnology, University of Massachusetts Chan Medical School, Worcester, MA 01605, USA.
* Correspondence to guocai.zhong@umassmed.edu (G.Z.); zhaojincun@gird.cn (J.Z.); yinyd@szbl.ac.cn (Y.Y.); liyujun@szu.edu.cn (Y.L.)

Mengjia Lu, Weitong Yao, Yujun Li, Danting Ma and Zhaoyong Zhang contributed equally to this work. Author order was determined by drawing straws.

The Supplementary Materials include:

Figs. S1 to S8

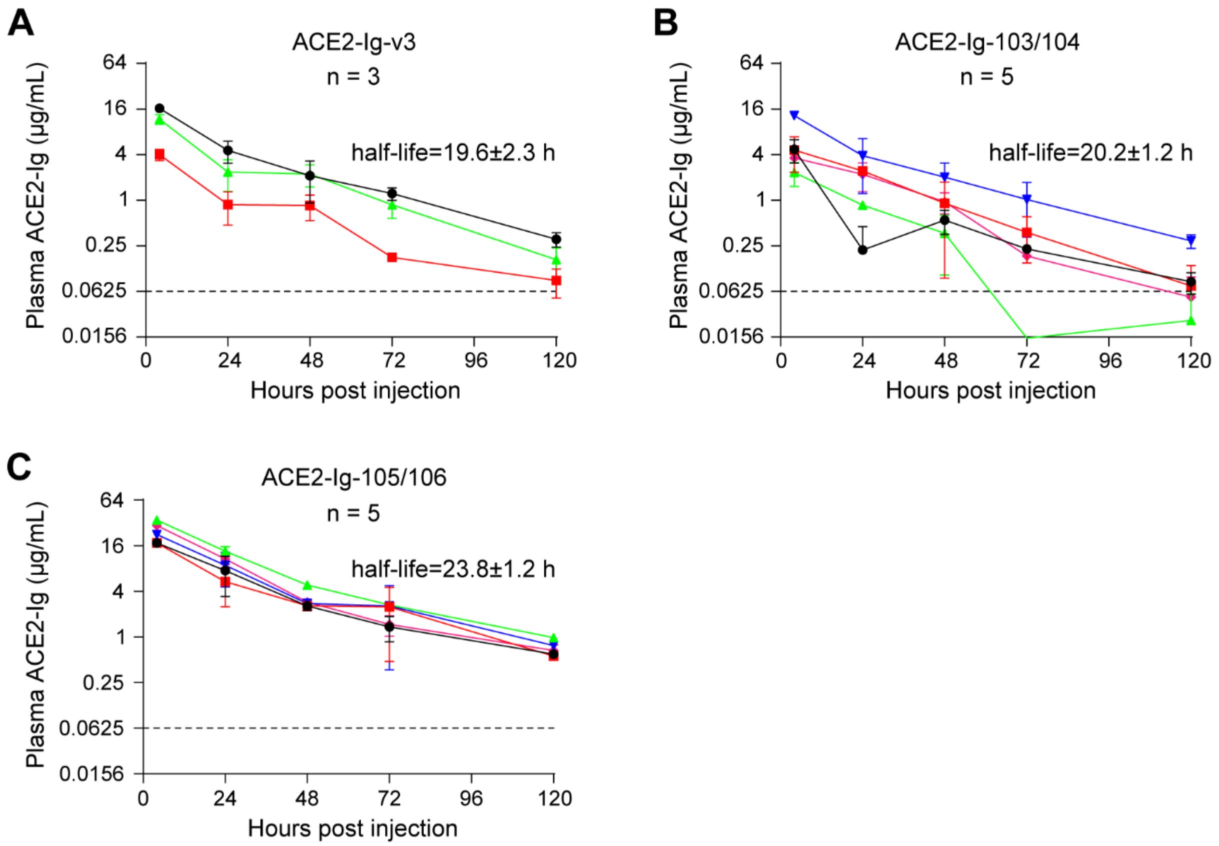

Fig. S1. Plasma half-life comparison of three related ACE2-Ig constructs.

(**A-C**) Female BALB/c mice were injected intraperitoneally (i.p.) with 14 mg/kg of ACE2-Ig-v3, -103/104, or -105/106 for protein half-life measurement. Blood samples were collected at the indicated time points and quantitative ELISA was performed to detect the corresponding ACE2-Ig proteins from plasma samples. Each solid line represents an animal. The dash lines represent the lower limit of detection of the quantitative ELISA assay.

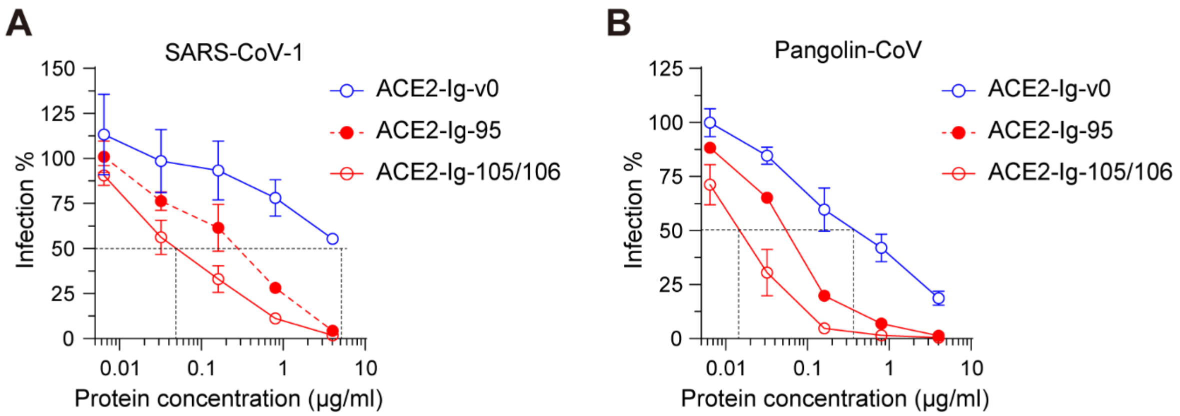

Fig. S2. Neutralization activities of three ACE2-Ig constructs against pseudoviruses of SARS-CoV-1 or a SARS-CoV-2-like coronavirus of pangolin origin (Pangolin-CoV).

HeLa-hACE2 cells, a stable cell line that overexpresses human ACE2, were infected with SARS-CoV-1 (A) or Pangolin-CoV (B) pseudovirus in the presence of the indicated inhibitor proteins. Pseudovirus infection-mediated luciferase reporter expression was measured at 48 hours post-infection. Luciferase signals observed at each inhibitor concentration were divided by the signals observed at concentration zero to calculate percentage-of-infection (Infection %) values. Data shown are representative of three independent experiments performed by two different people with similar results, and data points represent mean ± s.d. of three biological replicates.

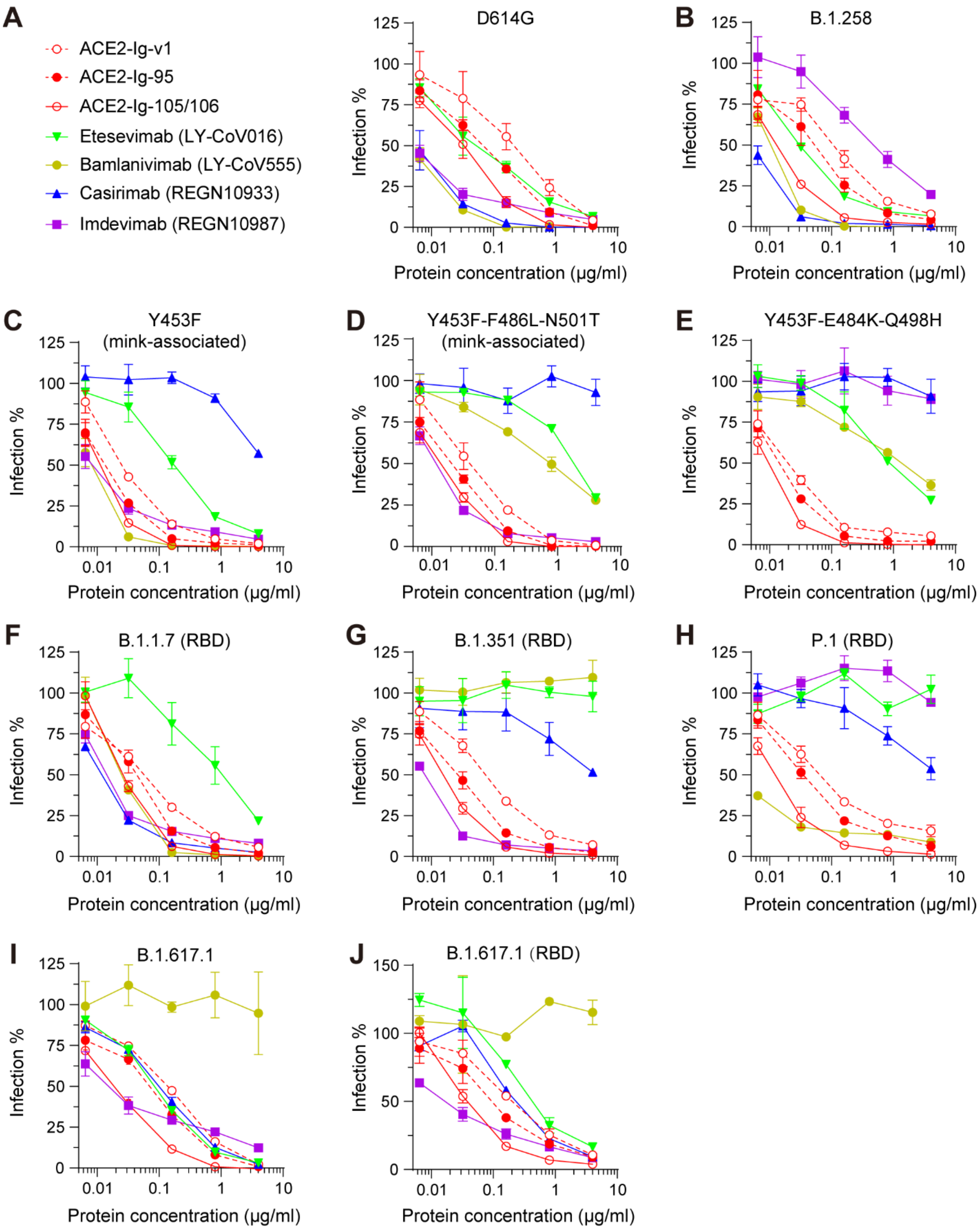

Fig. S3. ACE2-Ig-95 and -105/106 proteins but not monoclonal antibodies robustly neutralized pseudoviruses of diverse SARS-CoV-2 variants.

The indicated ACE2-Ig constructs were compared with four previously approved anti-SARS-CoV-2 monoclonal antibodies for their *in vitro* neutralization potencies against pseudoviruses of six

SARS-CoV-2 variants in HeLa-hACE2 cells, a stable cell line that overexpresses human ACE2. Pseudovirus infection-mediated luciferase reporter expression was measured at 48 hours post-infection. Luciferase signals observed at each inhibitor concentration were divided by the signals observed at concentration zero to calculate percentage-of-infection (Infection %) values. Data shown are representative of three independent experiments performed by two different people with similar results, and data points represent mean ± s.d. of three biological replicates.

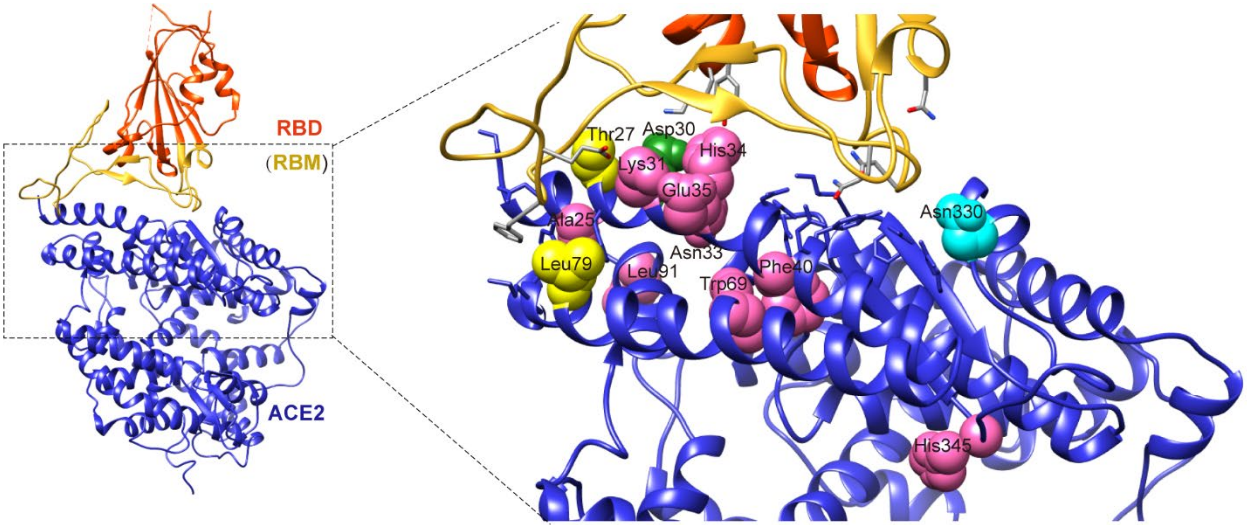

Fig. S4. Distribution of the mutated residues of the eight ACE2-Ig constructs.

Interactions between SARS-CoV-2 RBD (red) and ACE2 (blue) involve a large number of contact residues (PDB accession no. 6M0J). The receptor binding motif (RBM) within the RBD is indicated in yellow. All SARS-CoV-2 variant-associated RBD mutations investigated in this study are shown as sticks. ACE2-residues mutated in any of the eight ACE2-Ig constructs are shown as spheres and labelled. The green residue is Asp30 which was mutated to a Glu30 in the ACE2-Ig-v1.1 and ACE2-Ig-v3 constructs[20]. Yellow residues are Thr27 and Leu79, which were chosen for mutation by both Chan *et al*[27] and Glasgow *et al*[28]. The cyan residue, Asn330, was the third residue mutated in ACE2-Ig-Chan-v2.4 by Chan *et al*[27]. Pink indicates residues mutated in the four ACE2-Ig constructs (ACE2-Ig-Glasgow-293, -310, -311, and -313) developed by Glasgow *et al*[28].

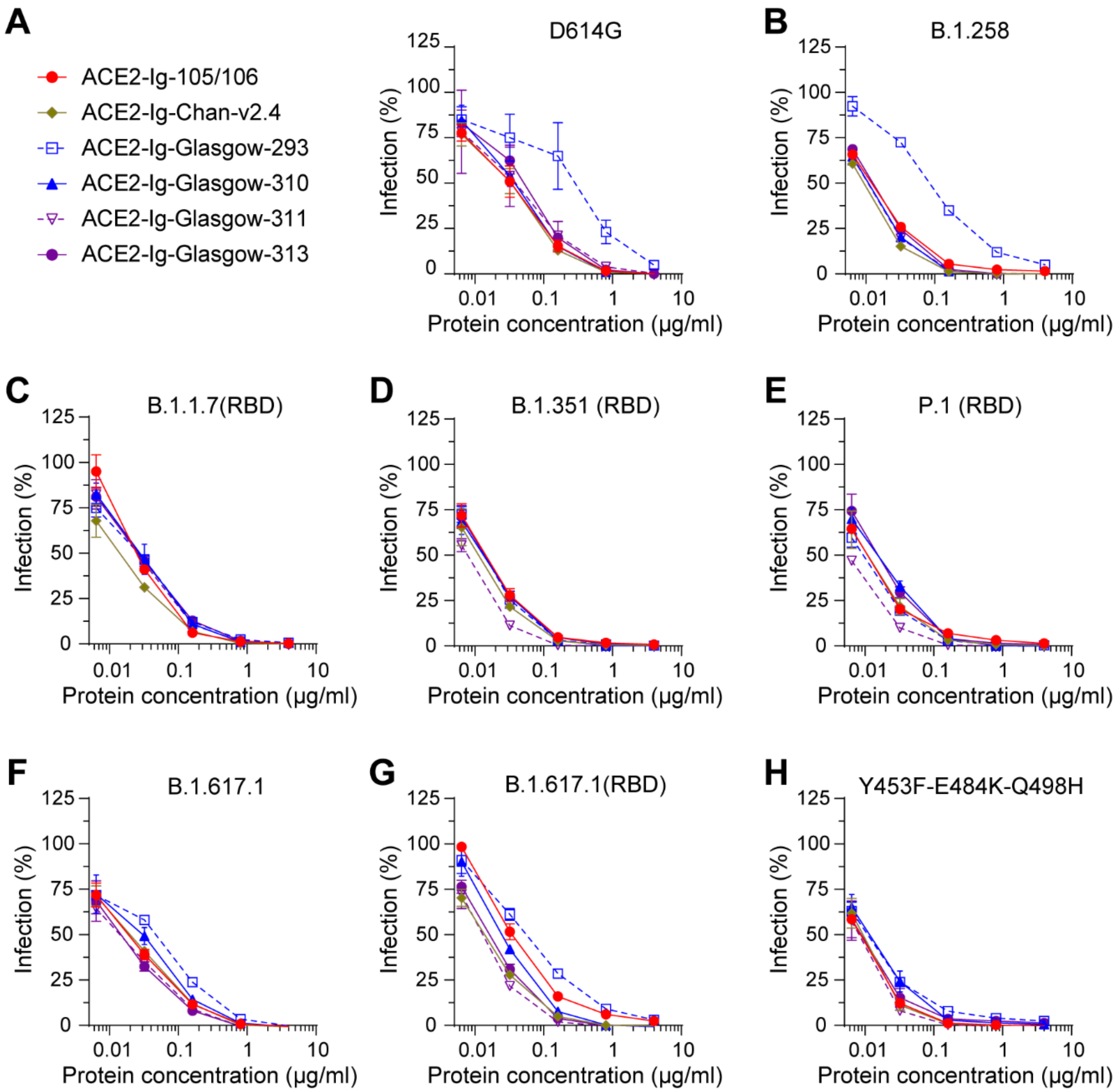

Fig. S5. A head-to-head comparison of ACE2-Ig-105/106 with five previously published ACE2-Ig constructs.

Pseudovirus neutralization experiments same as Fig 2 were performed to evaluate the neutralization potency and robustness of ACE2-Ig-105/106 and five surface-mutated dimeric soluble ACE2 constructs, including one from Chan *et al*[27], named here as ACE2-Ig-Chan-v2.4, and four from Glasgow *et al*[28], named here as ACE2-Ig-Glasgow-293, -310, -311, and -313. Data shown are representative of three independent experiments performed by two different people with similar results, and data points represent mean ± s.d. of three biological replicates.

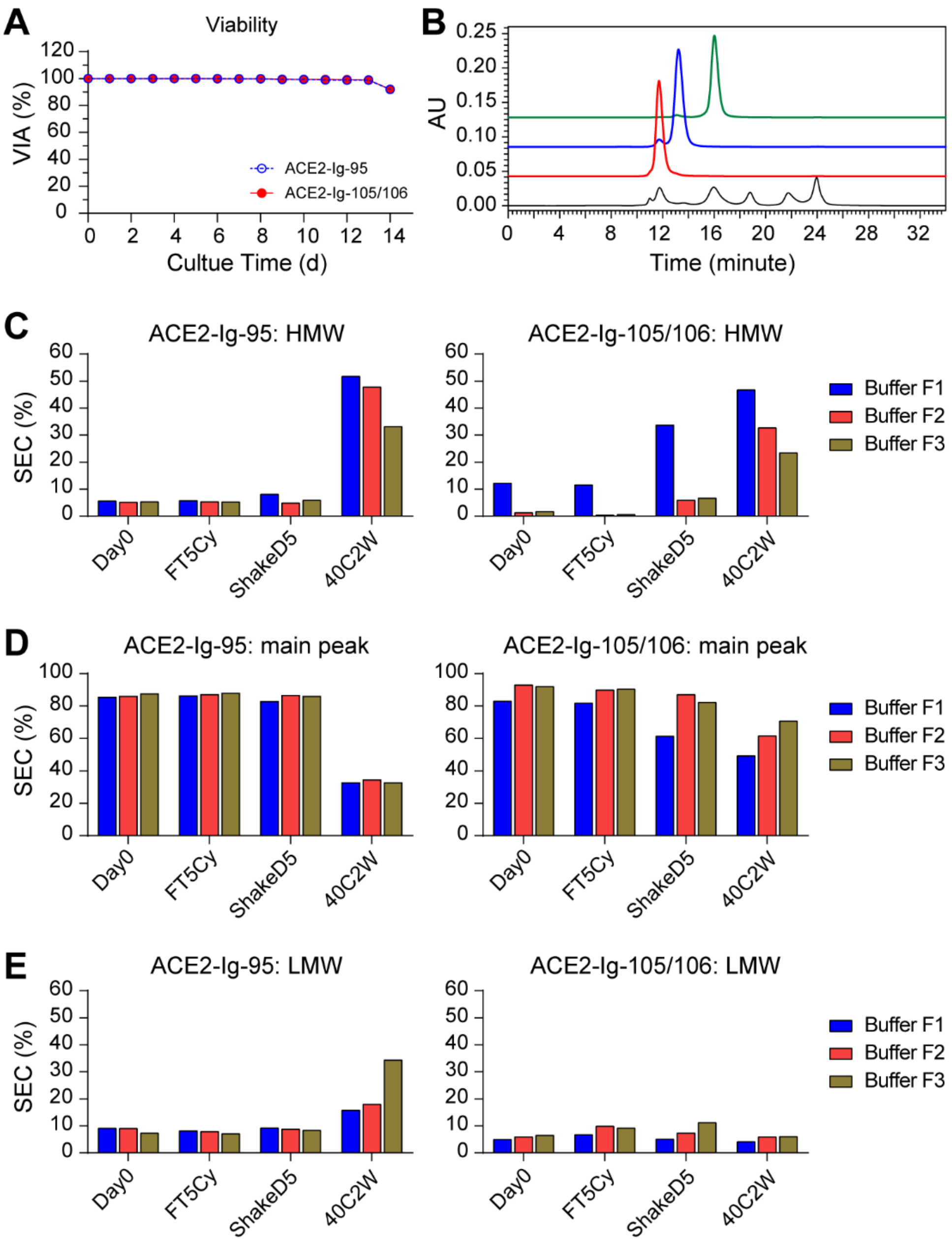

Fig. S6. ACE2-Ig-95 and ACE2-Ig-105/106 large-scale production and stress-condition stability tests.

(**A**) Two stable CHO cell pools that express ACE2-Ig-95 and ACE2-Ig-105/106, respectively, were generated and tested in a three-liter scale-up culture experiment. Cell viability was monitored throughout the 14-day culture period. (**B**) Purified ACE2-Ig-95 and ACE2-Ig-105/106 were analyzed by size exclusion chromatography. Green, an IgG1 antibody reference (145 kDa); blue, ACE2-Ig-95 (219 kDa); red, ACE2-Ig-105/106 (430 kDa); black, gel filtration standard. (**C-E**) Purified ACE2-Ig-95 and ACE2-Ig-105/106 proteins were prepared at 10 mg/mL concentration in three different buffers (F1, F2, and F3). All three buffers contain 40 mg/mL trehalose and 0.2 mg/mL polysorbate 80. In addition, buffer F1 (pH6.5) and F2 (pH7.0) have 10 mM Histidine. Buffer F3 (pH7.5) has 10 mM Tris-HCl. Proteins in these different buffers were then assessed for

their stability under the following three stress conditions: freeze-thaw stress (five cycles of freezing at -80 °C and thawing at room temperature; FT5Cy), shear stress (shaking at 300rpm, 37 °C, for one week; ShakeD5), and temperature stress (incubation at 40 °C for two weeks; 42C2W). Protein samples were then subjected to size exclusion chromatography (SEC) analysis to quantify the fractions of high molecular weight (B), main peak (C), and low molecular weight (D), respectively.

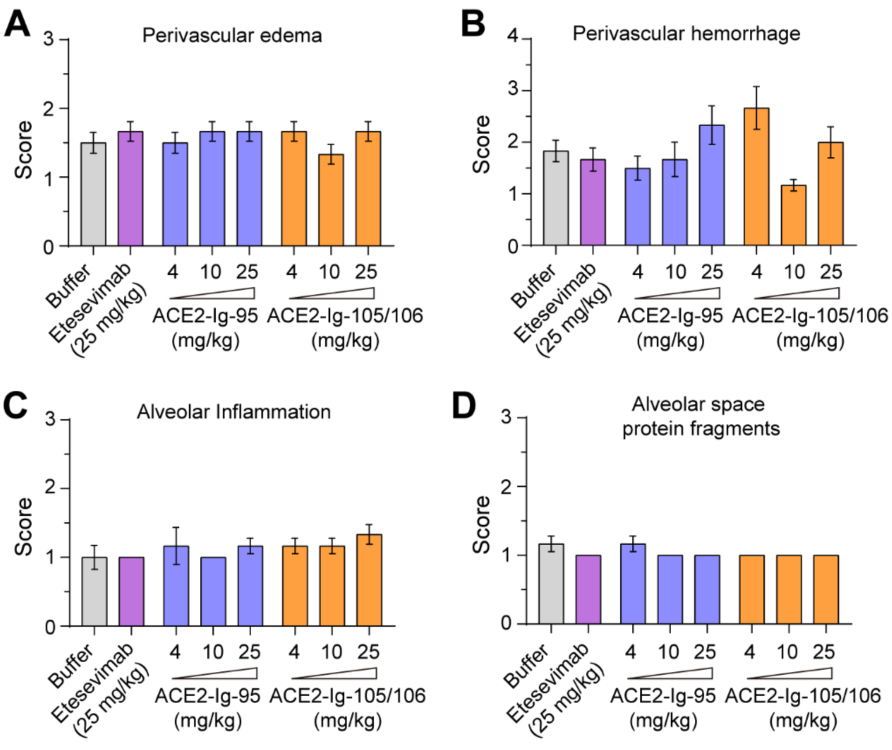

Fig. S7. Pathological score data for additional lung lesions analyzed in Fig 4D.

K18-hACE2 mice treated following the procedure in Fig 4A (n=6 per group) were sacrificed on day 5 post infection and the lungs were harvested for measuring viral load and histopathological changes. Data points here represents mean ± s.e.m of the pathological scores obtained from two lung tissue sections per animal, and six animals per group.

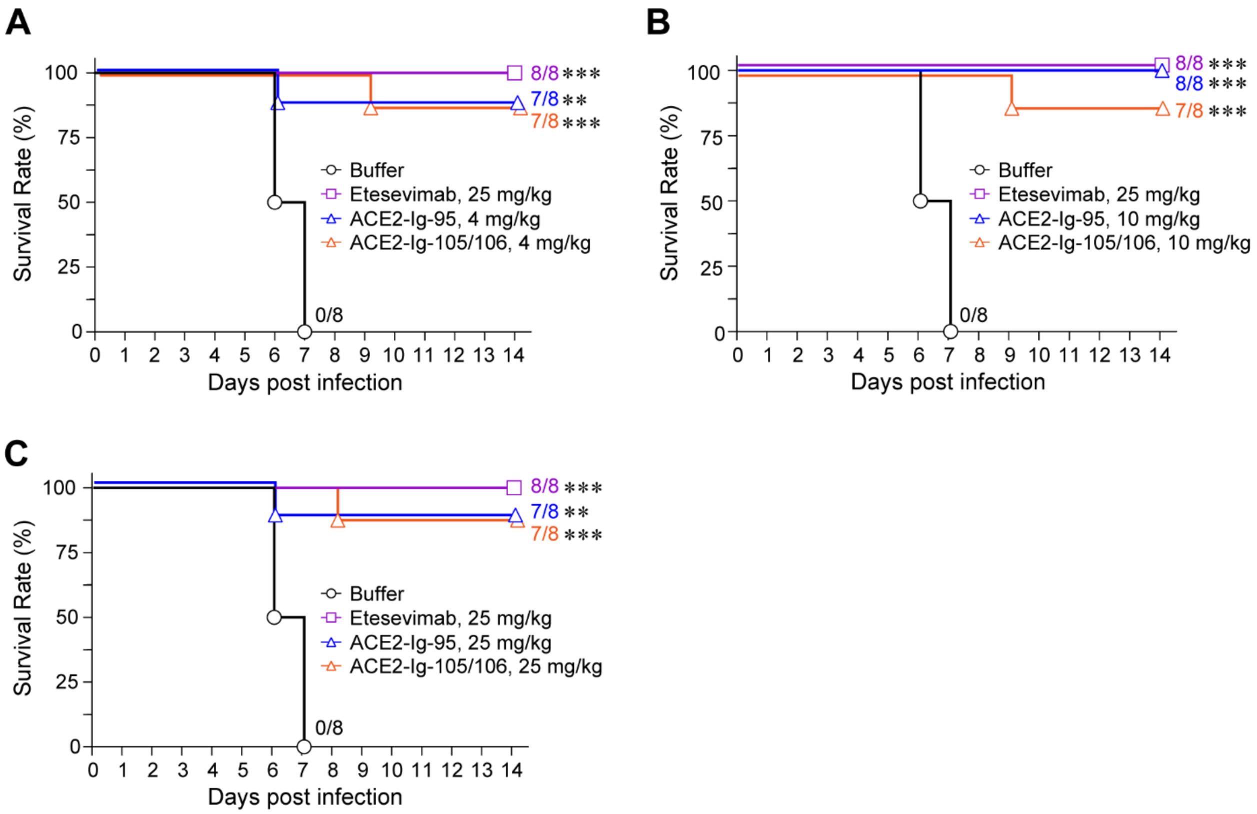

Fig. S8. ACE2-Ig-95 and ACE2-Ig-105/106 effectively protected K18-hACE2 mice from lethal SARS-CoV-2 infection.

Sixty-four SARS-CoV-2-infected K18-hACE2 mice were divided into eight treatment groups. Eight mice per group were treated daily for seven consecutive days with either buffer, etesevimab at 25 mg/kg, or ACE2-Ig-95 or ACE2-Ig-105/106 at 4, 10, or 25 mg/kg. Animals were continuously monitored from day 0 through day 14 post infection for survival and data were presented as Kaplan-Meier survival curves. Data for animals treated with ACE2-Ig-95 or ACE2-Ig-105/106 at each dose were plotted separately. The number of animals survived was indicated at the terminal point of each group. Log-rank test (Mantel-Cox) was performed to determine the statistical significance between the control and each treatment groups (**, $P<0.01$; ***, $P<0.001$).
